# Changes in Corporate Social Responsibility Efficiency in Chinese Food Industry Brought by COVID-19 Pandemic—A Study With the Super-Efficiency DEA-Malmquist-Tobit Model

**DOI:** 10.3389/fpubh.2022.875030

**Published:** 2022-05-09

**Authors:** Zhiting Chen, Wenzhong Zhu, Hainuo Feng, Houwei Luo

**Affiliations:** School of Business, Guangdong University of Foreign Studies, Guangzhou, China

**Keywords:** COVID-19, corporate social responsibility efficiency, super-efficiency DEA, Malmquist, Tobit model

## Abstract

Coronavirus disease 2019 (COVID-19) has brought about a significant and far-reaching impact on the world's business environment, corporations, and individuals. In the face of the general shortage of funds caused by the pandemic, assuming corporate social responsibility (CSR) is a problem for every enterprise manager. In the recent years, as corporate social responsibility (CSR) has become a hot topic globally, many enterprises have chosen to incorporate social responsibility into their development strategies. The food industry is a basic industry related to people's livelihood, especially in the pandemic. Its social responsibility efficiency deserves our attention. This article takes 17 sample enterprises whose CSR performance between 2012 and 2020 in China and reports are above the industry level as examples. Constructing the super-efficiency data envelopment analysis (DEA)-Malmquist-Tobit model explores the social responsibility efficiency of these enterprises. It analyzes the pandemic's impact on CSR efficiency. The results show that COVID-19 has promoted the social responsibility efficiency of the sample enterprises in the food industry. Besides, the level of technical efficiency and technological progress in the food industry is relatively high. Still, most enterprises are below the industry's average level. Before the outbreak of the pandemic, the size of enterprises is the key factor affecting the efficiency of CSR. After then, the listing years of enterprises then become the key factor.

## Introduction

The spread of coronavirus disease 2019 (COVID-19) has caused a serious economic and social crisis. Kimhi et al. (1) believe that the economic recession and increased economic uncertainty caused by the epidemic led to corporate bankruptcy or layoffs, which also increased the possibility of most individuals and families to face economic difficulties and economic pressure, causing social panic. For enterprises, on one hand, the sudden financial squeeze will drive companies to strategically pursue short-term returns, reduce or even give up corporate social responsibility investment, i.e., this crisis brings new challenges to all the enterprises in corporate social responsibility (CSR). On the other hand, the economic poverty and unemployment of the pandemic have put greater pressure on people, which caused anxiety, depression, and other psychological troubles. Thus, it is more urgent for enterprises to assume social responsibility, stop adding to the panic on the other side. In a way, it also puts pressure on enterprises to assume social responsibility (2).

For a long time, the food industry has been a basic industry related to the national economy and livelihood. Its healthy and stable development plays an important role in ensuring and improving people's living standards. At the same time, food enterprises are the main microsocial organizational carrier to effectively participate in public social governance and promote the modernization of national governance capacity. However, food safety is still an urgent social problem in China. In the face of most mixed food safety information, Chinese consumers often have negative emotions such as anxiety about food choices (3). This also means that current CSR situation in China's food industry is not optimistic. From the outbreak of “melamine” incident of the Sanlu Group in 2008 to the “expired meat” incident of Fuxi Shanghai branch in 2014 to the “Sudan red” duck egg incident in 2020 and the “Yi Di Xiang” food, toxic additive incident in 2021, food safety incidents emerge one after another. Therefore, these situations have aroused widespread public concern and thinking: whether food enterprises pay attention to social responsibility while developing themselves at present? Particularly, in front of the novel coronavirus pneumonia outbreak and other public health emergencies, what role do food enterprises play? And what kind of impact does COVID-19 have on the efficiency of food enterprises' social responsibility?

In June 2020, “Guidelines for the implementation of corporate social responsibility in the food industry” promulgated the social responsibility standards of the food industry. Although strengthening legislation can keep the moral bottom line of food enterprises, which is the main and effective measure to deal with the general lack of social responsibility of food enterprises in China, how to evaluate CSR while strengthening governance? Michael Porter, the father of the competitive strategy, once proposed that CSR is the cost and the source of opportunity, innovation, and competitiveness. CSR investment is a manifestation of corporate social responsibility and a strategic choice for enterprises to improve economic and social benefits. Conversely, the improvement of CSR level reflects the investment of CSR. It can be used to evaluate whether the investment of CSR reaches the expected effect, i.e., the investment efficiency of CSR. Then, studying the efficiency of CSR will help enterprises to make reasonable decisions on social responsibility, improve their ability to perform social responsibility, and even be willing to actively perform social responsibility because of the high efficiency of social responsibility investment. Therefore, this article selects 17 sample enterprises in the Chinese food industry from 2011 to 2020 as research samples and uses the super-efficiency data envelopment analysis (DEA)-Malmquist-Tobit model to study the investment efficiency and influencing factors of social responsibility of China's food industry listed companies; meanwhile, it explores the social responsibility performance of food production enterprises in the context of COVID-19.

The contribution of this article lies in the following three areas:

First, at present, most of the study on CSR mainly focuses on the influencing factors of CSR (4), behavior characteristics (5, 6), and behavior consequences (7, 8). But, few studies focus on a certain type of enterprise and pay attention to the impact of enterprise characteristics on CSR performance to evaluate its CSR efficiency. In addition, the food industry is listed as one of the key industries in the CSR report of the Chinese Academy of Sciences. Still, there is little research on the social responsibility performance of food enterprises. Therefore, this article focuses on food enterprises, combined with the current situation of China's food industry and the background of COVID-19, analyzes and performs the performance evaluation of CSR in China's food industry to fully reflect the efficiency of CSR in China's food enterprises, and improves the situation of China's food industry. So, it can enrich the areas of study and provide a new academic perspective.

Second, COVID-19 is a health event in the recent years. At present, study on the impact of COVID-19 mainly focuses on mental health (9) and economic and social levels (10). However, few studies have focused on the impact of COVID-19 on enterprises, especially their social responsibilities effectively. In fact, the emergence of COVID-19 has brought challenges to the performance of CSR in most industries, which has affected the input of CSR. But, because COVID-19 has been a health event in the recent years, few studies have focused on the impact of this new situation on food enterprises. Therefore, this article takes COVID-19 as the research background. First, based on the static analysis of the super-efficiency DEA model, we study the overall efficiency and ranking of the sample enterprises to get the overall CSR efficiency of the sample enterprises affected by the pandemic. Second, based on the dynamic analysis of the Malmquist productivity index method, this article studies the interannual total factor productivity change trend and its decomposition of sample enterprises. Finally, based on the Tobit regression analysis, this article studies the influencing factors of CSR efficiency in food enterprises to analyze the issue of CSR in the food industry more comprehensively and deeply. Therefore, this article will enrich the existing research on the CSR of food manufacturing enterprises. In addition, the background of COVID-19 also makes this study more epochal and practical.

Third, in the study of CSR efficiency, scholars mainly use two simple weight aggregation methods: first, scholars give equal weight to all the CSR categories (such as community relations, environmental performance, and human rights) (11) and assign the same weight by assuming that all the indicators have the same importance. However, this assumption is invalid in most cases (12); the second is to collect information about stakeholder preferences to assign weights to specific CSP categories (13). However, these aggregation methods have the following problems: because the composition, views, and preferences of stakeholders are dynamic, they do not have generally recognized weights in different situations (11, 12, 14); the total score lacks simple explanation; the weight does not represent the trade-off between CSP indicators; and when using different data sources, weights and total scores may lose their applicability and comparability (15). Therefore, this article creatively constructs the super-efficiency DEA-Malmquist model to evaluate the food industry's performance of social responsibility behavior. There are three main reasons for choosing those methods. First, the super-efficiency DEA is a nonparametric estimation method, which can measure the ratio of input and output without assuming the functional relationship between explanatory variables and explained variables, which can avoid the uncertainty of the relationship between CSR and business performance in the current academic research. The efficiency of food manufacturing enterprises in fulfilling corporate social responsibility can be measured from the ratio of input and output; second, the Malmquist index method can compare the input and output over time, measure the attention of Chinese food manufacturing enterprises to social responsibility, and the improvement of the efficiency of related resource allocation in the recent 10 years; finally, the Tobit model can analyze the key variables affecting the efficiency of corporate social responsibility based on the efficiency value and provide a practical reference for enterprises to improve corporate social responsibility.

Based on the above analysis, the remainder of this article is structured as follows: the second part is the study design, which is divided into three subsections of data sources, variable design and research methods, the third part is the empirical study, which is divided into the static analysis based on the CCR-based super-efficiency DEA model (CCR is a kind of data envelope model, which was created by A. Charnes & W. W. Cooper & E. Rhodes.), dynamic analysis based on the Malmquist productivity index method and analysis of factors influencing CSR efficiency based on the Tobit model, and finally, the section is a recommendation to the government, food industry, and enterprises based on the conclusion.

## Research Design

### Data Source

In this article, 17 companies with the top CSR ratings in the food industry in China's national CSR reports from 2011 to 2020 were selected for this study. The basic information and related data are obtained from the official annual reports of the enterprises and the China Stock Market and Accounting Research (CSMAR) Database. The proximity interpolation method processed missing values to ensure data completeness and accuracy. In this article, Excel 2016 is used to organize the data initially and MaxDEA version 8.21 and Stata version 15.0 are used to do relevant empirical study on the sample data.

### Variable Design

Based on the idea of “input-output,” this article regards the enterprise as an economic organization with the responsibility of stakeholders for inputs and outputs. It takes the maximization of enterprise value as the financial objective of the enterprise. On this basis, relevant indicators are selected as follows, which are shown in Table 1.

**Table 1:** Input-output index system of corporate social responsibility (CSR) in food industry.

**Variables**	**Stakeholders**	**Indicators**	**Calculation formula**
Inputs	Shareholder	Earnings per share	Net income/Number of common shares
	Employees	Employee profitability level	Cash paid to and for employees/Total operating income
	Creditors	Gearing ratio	Total liabilities/Total assets
	Consumers	Operating cost ratio	The total cost of operations/Total operating revenue
	Government	Tax contribution rate	(Taxes paid - Tax refunds received)/Total operating income
Outputs	Enterprise	Return on net assets	Net Income/Average Net Worth
		Tobin Q	The market value of the total capital of the enterprise/Replacement cost of the total capital of the enterprise

*Source: Literature Research*.

In terms of input indicators, drawing on the study results of related scholars and considering the availability of indicators and the basic requirements of super-efficiency DEA, the following five variables were selected to reflect the degree of social responsibility fulfillment of listed companies as explanatory variables: earnings per share (X_1_), income per employee (X_2_), cash flow ratio (X_3_), cash received from sales ratio (X_4_), and tax contribution ratio (X_5_).

In terms of output indicators, for most firms, the most original motivation for fulfilling social responsibility lies in the legal obligations and expected benefits of CSR (16). In the long run, the active fulfillment of CSR can improve corporate performance. In the short run, fulfilling responsibility to different stakeholders has different effects on business performance. Therefore, this article selects the return on net assets and Tobin's Q as the output indicators of CSR. The return on net assets, which directly reflects the profitability level of listed companies, can measure the financial industry's ability to use its resources to obtain profits. Compared with the accounting rate of return, Tobin's Q is considered to have the following advantages. It reflects its expected future profits, contains an automatic adjustment for risk, and is less sensitive to inflation (17–19). Due to the wide support of western theoretical models and empirical evidence, Tobin's Q has also gradually become one of the main measures of firm value in empirical studies.

### Descriptive Statistics

Earnings per share, per capita income of employees, cash flow ratio, cash received from sales ratio, and tax contribution rate are taken as input variables and the return on net assets and Tobin's Q are taken as output variables. The descriptive statistical results of each variable are shown in Table 2.

**Table 2:** Results of descriptive statistics of variables.

**Variable**		**Mean**	**Std. Dev**.	**Min**	**Max**	**Observations**
Return on net assets	Overall	0.141	0.128	−0.308	0.500	*N* = 170
	Between		0.115	0.002	0.348	*n* = 17
	Within		0.062	−0.180	0.353	T = 10
Tobin Q	Overall	2.462	1.702	0.564	11.976	*N* = 170
	Between		1.334	1.062	5.658	*n* = 17
	Within		1.101	−0.589	8.806	T = 10
Shareholder	Overall	1.998	5.085	−0.740	37.170	*N* = 170
	Between		4.651	0.032	19.448	*n* = 17
	Within		2.321	−9.010	19.720	T = 10
Employees	Overall	115609.400	68280.600	31445.260	407298.600	*N* = 170
	Between		61414.130	58128.650	287061.700	*n* = 17
	Within		33036.410	5865.077	235846.300	T = 10
Creditors	Overall	0.350	0.303	−0.267	1.251	*N* = 170
	Between		0.265	0.034	1.030	*n* = 17
	Within		0.159	−0.143	0.851	T =10
Consumers	Overall	1.109	0.097	0.740	1.524	*N* = 170
	Between		0.066	0.915	1.196	*n* = 17
	Within		0.073	0.895	1.490	T = 10
Government	Overall	0.099	0.073	0.005	0.293	*N* = 170
	Between		0.071	0.018	0.222	*n* = 17
	Within		0.024	0.038	0.199	T =10

*Source: Data Processing*.

### Research Methodology

#### CCR-Based Super-Efficiency Data Envelopment Analysis Model

Data envelopment analysis is a relative efficiency evaluation method proposed by American operations researcher Charnes et al. (20) and other scholars. Its basic principle is to use a linear programming model to evaluate the relative effectiveness among decision units with multiple inputs and outputs. It is one of the most commonly used nonparametric frontier efficiency analysis methods. This article analyzes the inputs and outputs of CSR from the perspective of constant returns to scale.

**Figure 1:**
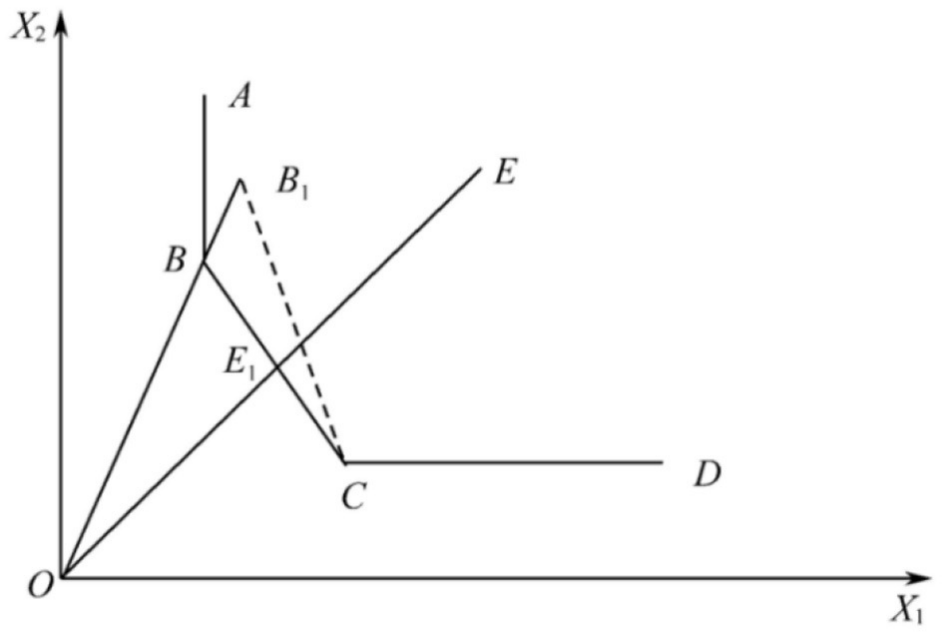
Input-oriented super-efficiency data envelopment analysis (DEA).

When using traditional DEA to evaluate the efficiency of decision units, multiple decision units may be in the production frontier simultaneously, resulting in multiple decision units being relatively efficient and, thus, unable to determine which is better or worse. To remedy this shortcoming, Andersen and Petersen (21) established an input-oriented super-efficiency DEA model, which enables the comparison of efficiency among relatively efficient decision units, overcoming the shortcoming that the traditional DEA model cannot make further evaluation and comparison of multiple decision units and enabling the comparison and ranking of efficient decision units. The specific principle is shown in Figure 1. When calculating the efficiency value of point B, it is excluded from the reference set of the decision unit. The effective production frontier is changed from ABCD to ACD and the efficiency value of point B becomes OB_1_/OB > 1. While point E, the original DEA invalid, still has ABCD as its production frontier, the evaluation value is still OE_1_/OE <1 in line with the traditional DEA model.

Its linear programming equation can be expressed as follows. Assuming that there are *n* decision units and *s* outputs are obtained using *m* input variables, the linear programming equation is:


(1) $$\left\{ \begin{array}{l} \;\min[\theta -\varepsilon (\Sigma^{\underset{m}{i=1}}s_{i}{\;}^{-}+\Sigma^{\underset{s}{i=1}}s_{i})]\\ \;s.t.\Sigma^{\underset{n}{i=1,j\neq k}}X_{ij}\lambda_{j}+s_{i}{\;}^{-}\leq \theta X_{0}\;{\;}_{\left(\right)}\;\\ \;\Sigma^{\underset{n}{i=1,j\neq k}}Y_{j}\lambda_{j}-s_{r}{\;}^{+}=Y_{0}\\ \lambda_{j}\geq 0,j=1,2,...,n,s_{r}{\;}^{+}\geq 0,s_{r}{\;}^{-}\geq 0 \end{array}\right.$$

Where *X* and *Y* denote input and output matrices, λ is a constant, $\varepsilon {{{s_{i}}^{+}s}_{i}}^{-}$ denote slack variables, and θ denotes the efficiency value. When θ > 1, it means the decision unit is technically effective. When θ < 1, it is in an ineffective state.

#### Malmquist Index

To more accurately evaluate the temporal trend of the decision unit and explore the causes, further analysis can be done using the Malmquist productivity index. Total factor productivity (TFP) is derived by comparing the technological distance functions of the two periods: the geometric mean of TFP for the two periods is calculated and the total efficiency can be decomposed into the technical change (TECHCH) and the technical efficiency change (EFFCH). Technical efficiency change can be further decomposed into pure efficiency change (PECH) and scale efficiency change (SECH). Technical efficiency change (EFFCH) reflects whether the industrial structure can meet the overall requirements and increase the economic and social benefits. It indicates the change in the efficiency of enterprises on CSR of food enterprises. If the index is >1, it indicates an increase in efficiency of resource allocation relative to the previous period and if the index is <1, the efficiency decreases. If the index is equal to 1, it indicates no change in efficiency. The technical change (TECHCH) is used to measure the efficiency of CSR inputs on the output of science and technology innovation. If the index is >1, it indicates that CSR commitment has contributed to technological innovation. If the index is <1, the technology is regressing. Pure efficiency change (PECH) is a measure of the role of firms in science and technology innovation (STI) output due to management, technology, and financial inputs, under the condition that the scale of the firm's inputs to stakeholders remains constant. If the index is >1, it indicates that corporate social responsibility drives the improvement of STI output. If it is <1, it indicates that CSR causes the decrease of STI output. If it is equal to 1, then CSR undertakes no effect on STI. Scale efficiency change (SECH) is used to measure the scale of CSR undertaking due to the factor of enterprise scale affecting the efficiency of enterprise STI. The relationship among them is as follows:


(2) $$\begin{array}{r} TFPCH=TECHCH*PECH*SECH \end{array}$$

The decomposition of the input-based Malmquist TFP index from period *s* to period *t* is follows:


(3) $$\begin{array}{l} m_{i}(q_{s},q_{t},x_{s},x_{t})={\left[m^{s}{\;}_{i}\left(q_{s},q_{t},x_{s},x_{t}\right)\times m^{t}{\;}_{i}\left(q_{s},q_{t},x_{s},x_{t}\right)\right]}^{\frac{1}{2}}\;\\ \;={\left[d^{s}{\;}_{i}\left(q_{s},q_{t},x_{s},x_{t}\right)\times d^{t}{\;}_{i}\left(q_{s},q_{t},x_{s},x_{t}\right)\right]}^{\frac{1}{2}} \end{array}$$

In formula (3), *m*_*i*_ represents the total factor productivity (TFP), ${d^{s}}_{i}$ and ${d^{t}}_{i}$ denote the input-oriented distance functions in periods *s* and *t*, *q*_*s*_and *q*_*t*_denote the output in the corresponding periods *s* and *t*, and *x*_*s*_ and *x*_*t*_ denote the input in the corresponding period.

Organizing the above equation yields:


(4) $$\begin{array}{l} m_{i}\left(q_{s},q_{t},x_{s},x_{t}\right)=d^{t}{\;}_{i}(x_{t},q_{t})/d^{s}{\;}_{i}(x_{s},q_{s})[d^{s}{\;}_{i}(x_{t},q_{t})/d^{t}{\;}_{i}(x_{t},q_{t})\\ \;\times d^{s}{\;}_{i}\left(x_{s},q_{s}\right)/d^{t}{\;}_{i}(x_{s},q_{s}){]}^{1/2} \end{array}$$

The first half of the formula ${d^{t}}_{i}\left(x_{t},q_{t}\right)/{d^{s}}_{i}\left(x_{s},q_{s}\right)$ represents the change in technical efficiency from period s to period t; the second half of the formula represents the change in technical progress in the corresponding period. The relative technical efficiency change (EFFCH) index reflects the change in relative efficiency under the conditions of free factor disposition and constant returns to scale. When the change in technical efficiency is larger than 1, it indicates an increase in relative technical efficiency. The value is independent of the selection of the reference period. The change in technical progress (TECHCH) reflects the movement of the production frontier surface between the two periods. It represents technological innovation and the high or low of this value is related to selecting the reference period. When the change in technological progress is larger than 1, it means that the production frontier is moving up. The change in technical efficiency can be further decomposed into the change in pure technical efficiency and scale efficiency. The change in technical efficiency equals the multiplication of pure technical efficiency and scale efficiency.

#### Tobit Model

Tobit model, which is created by Tobin in 1958, also known as the sample selection model, belongs to a regression model in which the dependent variable is restricted. Its concept was first proposed by James Tobin, a Nobel Prize winner in Economics. Then, a large number of economists have continuously optimized it. With the continuous improvement and maturity of the method, especially in the study of restricted data, the Tobit model based on the principle of maximum likelihood estimation has been widely used in various fields of study because it can avoid large bias in the values taken by the explanatory variables due to the satisfaction of certain constraints. The super-efficiency DEA and Malmquist index can objectively reflect the social responsibility efficiency of sample enterprises. Still, it cannot explore the key influencing factors of CSR efficiency. In addition, in this article, it is reasonable and feasible to use the Tobit model because CSR efficiency takes values above 0, which is consistent with the restricted situation of the accepted variables in the Tobit regression.

Therefore, Tobit regression is selected to carry out further study on them. In this section, the Tobit regression model with the random and fixed effects is used to explore the main factors affecting CSR efficiency in the food industry and the comparison of the influencing factors before and after the occurrence of the pandemic to provide some theoretical basis for the enterprises' improvement of CSR efficiency. CSR efficiency measured by the super-efficiency DEA model in the previous section is used as the explanatory variable. The panel data of food enterprises from 2011 to 2020 are selected as the sample.

## Empirical Study

### Static Analysis Based on the Super-Efficiency Data Envelopment Analysis Model

Based on the super-efficiency DEA model, this article uses MaxDEA version 8.21 to measure the social responsibility input and output data of the 17 sample enterprises in the food industry from 2011 to 2020 to obtain their efficiency value and ranking. The results are shown in Table 3.

**Table 3:** The super-efficiency data envelopment analysis (DEA) efficiency values of the 17 sample enterprises from 2011 to 2020.

**DMU**	**2011**	**2012**	**2013**	**2014**	**2015**	**2016**	**2017**	**2018**	**2019**	**2020**	**Average**	**Ranking**
Chengde Lolo	1.792	1.963	1.969	2.435	1.571	1.627	1.672	1.730	1.650	1.396	1.780	6
Wuliangye	0.820	0.837	1.014	0.739	0.508	0.575	0.918	0.691	1.114	1.437	0.865	14
New Hope	3.246	2.745	2.128	1.174	1.163	1.440	1.116	1.466	1.985	1.294	1.776	7
Royal Group	1.422	0.835	0.766	1.159	1.179	1.402	0.922	2.019	1.670	5.106	1.648	8
Sanyuan	3.317	1.411	6.787	1.498	1.023	0.689	6.874	0.668	0.697	0.681	2.365	3
Tongwei	1.639	2.070	2.123	2.033	1.938	1.171	1.572	1.528	1.271	3.070	1.842	4
Guizhou Maotai	1.058	1.221	1.210	0.874	0.990	0.831	1.613	1.401	1.948	1.291	1.244	11
Bright Dairy	1.091	0.589	0.777	0.759	0.824	0.663	0.860	0.917	0.764	0.646	0.789	15
Tsingtao Beer	0.684	0.518	0.491	0.581	0.837	0.492	0.444	0.519	0.488	0.548	0.560	16
Erie Shares	1.240	1.548	1.042	1.540	1.301	1.261	1.260	1.260	1.265	1.094	1.281	10
Yanjing Beer	0.569	0.497	0.585	0.539	0.461	0.405	0.483	0.611	0.522	0.711	0.538	17
Shuanghui Development	1.639	1.527	1.892	1.284	1.272	1.835	2.128	2.570	1.663	2.079	1.789	5
Sanquan Food	0.959	0.616	1.179	0.761	2.672	0.937	0.852	0.794	1.224	1.215	1.121	12
Yanghe Shares	1.466	1.717	1.034	0.864	1.143	0.982	0.877	0.814	0.754	1.276	1.093	13
Delisi	0.992	0.807	1.123	0.928	3.104	4.014	6.672	2.417	6.606	1.366	2.803	2
Vivian Shares	2.245	7.653	1.498	1.063	4.835	1.471	0.728	1.117	10.373	1.580	3.256	1
COFCO Sugar	0.840	2.020	0.906	2.076	1.556	2.739	0.773	0.905	0.817	0.729	1.336	9
Average	1.472	1.681	1.560	1.194	1.552	1.326	1.751	1.260	2.048	1.501	1.534	–

*Source: Data Processing*.

In Table 3, from the overall results, average CSR efficiency value of the sample enterprises from 2011 to 2020 is 1.534 and the overall level of CSR efficiency value is high.

From the perspective of individual enterprises, the results of ranking the social responsibility efficiency value of the sample enterprises are: Vivian Shares > Delisi > Sanyuan > Tongwei > Shuanghui Development > Chengde Lolo > New Hope > Royal Group > Erie Shares > Guizhou Maotai > Sanquan Food > Yanghe Shares > Wuliangye > Bright Dairy > Qingdao Beer > Yanjing Beer. The average CSR efficiency value of Vivian Shares in the past 10 years is 3.256 and that of Yanjing Beer is 0.538, with a difference of 2.718 units. It shows a significant gap among enterprises with good CSR performance in the food industry.

Other enterprises reached an efficient state from 2011 to 2020, except Wuliangye, Bright Dairy, and Tsingtao Beer. This issue indicates that the enterprises with good CSR performance in the food industry still have inefficient CSR inputs, so the efficiency of CSR in the food industry needs to be improved.

To further analyze CSR efficiency of the food industry under COVID-19, this article compares the efficiency of 17 sample enterprises in 2019 and 2020, as shown in Table 4. In 2020, the social responsibility efficiency values of 10 enterprises, namely, Chengde Lolo, New Hope, Sanyuan, Guizhou Maotai, Bright Dairy, Erie Shares, Sanquan Food, Delisi, and Vivian Shares, all showed a significant downward trend. Among them, the efficiency value of Vivian Shares decreased the most, by 84.77%. Delisi took second place, down 79.32%, and they fell from no. 1 to no. 4 and no. 2 to no. 7, respectively. At the same time, CSR efficiency value of Wuliangye, Royal Group, Tongwei, Tsingtao Beer, Yanjing Beer, Shuanghui Development, and Yanghe Shares showed an upward trend. Among them, Royal Group and Tongwei increased significantly by 205.77 and 141.51%. Their ranking rises from no. 5 to no. 1 and no. 8 to no. 2. Overall, the average CSR efficiency of 17 sample enterprises in 2020 increased by 13.44% compared with 2019. Thus, it can be seen that the pandemic has generally promoted the assumption of CSR. There are individual differences in the impact on the food industry, which hit the enterprises with better CSR performance before the pandemic.

**Table 4:** Comparison of CSR efficiency values of 17 sample enterprises in 2019 and 2020.

**DMU**	**2019**	**Sort by**	**2020**	**Sort by**	**Range of change**
Chengde Lolo	1.650	7	1.396	6	−15.39%
Wuliangye	1.114	11	1.437	5	28.97%
New Hope	1.985	3	1.294	8	−34.82%
Royal Group	1.670	5	5.106	1	205.77%
Sanyuan	0.697	15	0.681	15	−2.26%
Tongwei	1.271	8	3.070	2	141.51%
Guizhou Maotai	1.948	4	1.291	9	−33.74%
Bright Dairy	0.764	13	0.646	16	−15.34%
Tsingtao Beer	0.488	17	0.548	17	12.25%
Erie Shares	1.265	9	1.094	12	−13.51%
Yanjing Beer	0.522	16	0.711	14	36.29%
Shuanghui Development	1.663	6	2.079	3	25.03%
Sanquan Food	1.224	10	1.215	11	−0.71%
Yanghe Shares	0.754	14	1.276	10	69.23%
Delisi	6.606	2	1.366	7	−79.32%
Vivian Shares	10.373	1	1.580	4	−84.77%
COFCO Sugar	0.817	12	0.729	13	−10.74%
Average	2.048	–	1.501	–	13.44%

*Source: Data Processing*.

### Dynamic Analysis Based on the Malmquist Productivity Index Method

Based on the static analysis of the CSR efficiency of the sample enterprises, this article further measured the Malmquist index of the sample enterprises from 2011 to 2020 to dynamically analyze their CSR efficiency. The MaxDEA version 8.21 was used to calculate the technical efficiency change (EFFCH), technical change (TECHCH), pure efficiency change (PECH), scale efficiency change (SECH), and total factor productivity (TFP) change of the 17 sample enterprises in the food industry from 2011 to 2020.

**Figure 2:**
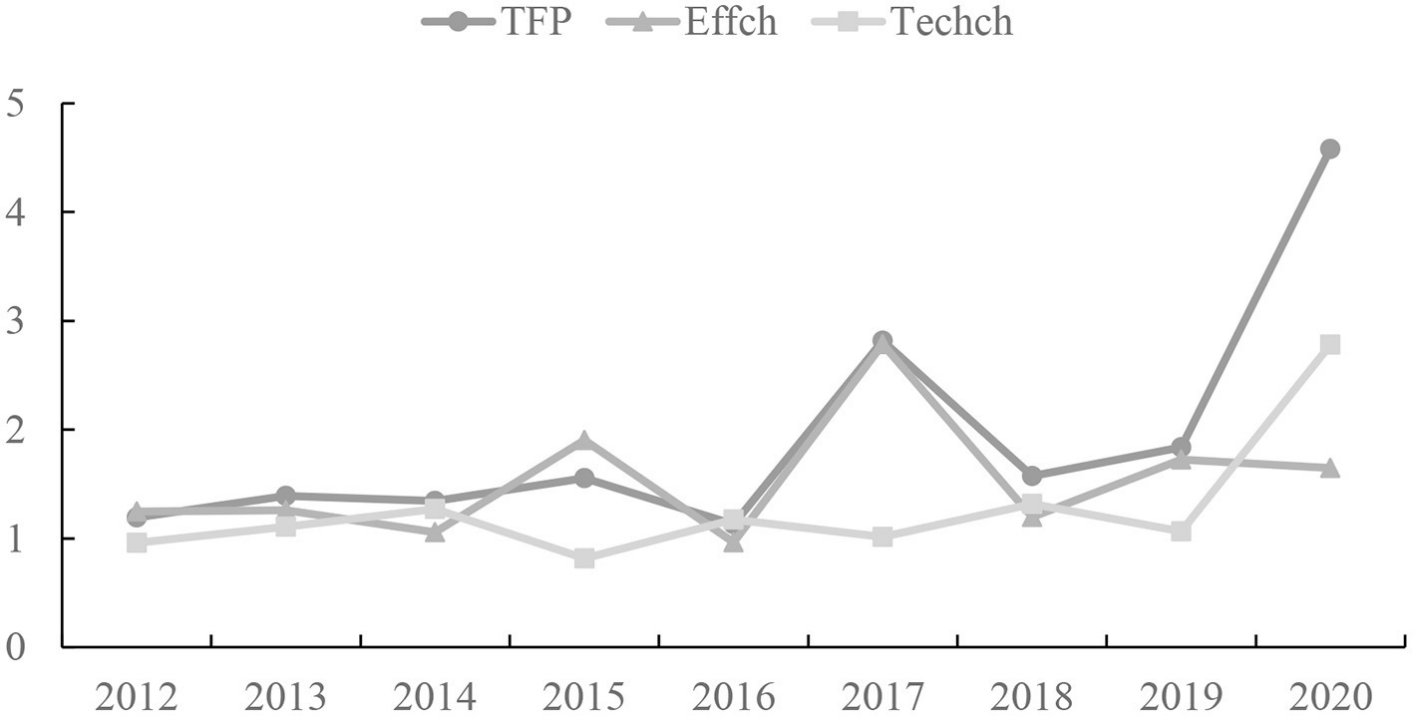
The annual change trend of total factor productivity (TFP), technical efficiency change (EFFCH), and technological progress change (TECHCH) of 17 sample enterprises in the food industry from 2011 to 2020.

The total factor productivity and its decomposition of the sample firms are shown in Table 3. From Table 5, it can be seen that, from the overall situation, the average total factor productivity from 2011 to 2020 is 1.957 and the annual total factor productivity is larger than 1. The overall CSR efficiency is on the rise year by year. Among them, the total factor productivity in 2016 was the lowest, at 1.130, and the total factor productivity in 2020 is the highest, at 4.581. It shows a large gap among years in the total factor productivity of the sample enterprises' social responsibility. The comprehensive efficiency of the sample enterprises' social responsibility in the food industry under the pandemic has reached the maximum in the past 10 years. The trends of technical efficiency change (EFFCH), technical change (TECHCH), and total factor productivity (TFP) change of each enterprise from 2011 to 2020 are represented by line graphs, as shown in Figure 2.

**Table 5:** Annual total factor productivity (TFP) index and its decomposition of social responsibility efficiency of 17 sample enterprises in the food industry from 2011 to 2020.

**Year**	**Total factor productivity (TFP)**	**Pure technical efficiency** **(Pech)**	**Scale efficiency (Sech)**	**Technical efficiency** **(Effch)**	**Technological advances (Techch)**
2012	1.193	1.294	0.962	1.245	0.958
2013	1.392	1.126	1.118	1.259	1.106
2014	1.343	1.157	0.914	1.058	1.269
2015	1.552	1.881	1.011	1.903	0.816
2016	1.130	1.003	0.961	0.964	1.173
2017	2.818	1.607	1.734	2.786	1.012
2018	1.574	0.989	1.209	1.195	1.318
2019	1.836	2.032	0.849	1.725	1.065
2020	4.581	1.405	1.172	1.647	2.782
Average	1.957	1.388	1.103	1.532	1.278

*Source: Data Processing*.

Pure efficiency change and SECH have different degrees of influence on technical efficiency in different years. In terms of EFFCH from Table 5 and Figure 2, it can be seen that the technical efficiency of enterprises fluctuated greatly in the past 10 years. Among them, only the technical efficiency in 2016 was <1, indicating that its use of technical factors is less efficient, which shows that the application of technical factors in sample enterprises reaches a high level. In a word, the average technical efficiency is rising. Still, the average scale efficiency pulls down the level of technical efficiency growth.

From the perspective of TECHCH, the technological progress in 2012 and 2015 was <1 and that in other years was more than 1. In the rising state, the average technological progress efficiency is 1.278, indicating that all the enterprises have performed well in applying new technologies and developing new products in CSR.

On the whole, technological progress is the main factor affecting the change in total factor productivity of CSR and the food industry has the characteristic of constantly paying attention to technology and carrying out reforms in production technology. The trend of technological progress change is generally more consistent with the change of total factor productivity. As shown in Figure 2, the growth of technological progress in 2020 is larger. Therefore, in the context of the pandemic, enterprises should pay more attention to technological progress, introduce new technologies, improve their innovation capabilities, and actively respond to the challenges of global and systemic risks.

To analyze the total factor productivity index of social responsibility efficiency and its decomposition of sample companies under the pandemic, this article collated the comprehensive efficiency index of social responsibility and its decomposition index of each enterprise sample company in 2020, as shown in Table 6. Table 6 shows six enterprises with a TFP index of <1, namely, New Hope, Royal Group, Sanyuan, Bright Dairy, Shuanghui Development, and COFCO Sugar. The total factor productivity of these six enterprises showed a downward trend in 2020. Combined with the static performance in 2020, it is found that the operation effect of most enterprises is not ideal, except Huang Group, once again indicating that the pandemic had a serious impact on the operations of companies in the food industry.

**Table 6:** TFP index and its decomposition of social responsibility efficiency of sample companies in the 17 sample enterprises in the food industry in 2020.

**Serial** **number**	**DMU**	**Total factor productivity** **(TFP)**	**Pure technical efficiency (Pech)**	**Scale efficiency** **(Sech)**	**Technical efficiency (Effch)**	**Technological advances** **(Techch)**
1	Chengde Lolo	1.007	0.786	1.076	0.846	1.190
2	Wuliangye	2.083	2.035	0.634	1.290	1.615
3	New Hope	0.643	0.303	2.154	0.652	0.986
4	Royal Group	0.946	4.689	0.652	3.058	0.309
5	Sanyuan	0.683	0.914	1.070	0.977	0.699
6	Tongwei	2.759	5.393	0.448	2.415	1.142
7	Guizhou Maotai	1.356	0.772	0.858	0.663	2.046
8	Bright Dairy	0.961	0.978	0.866	0.847	1.136
9	Tsingtao Beer	1.311	1.074	1.045	1.123	1.168
10	Erie Shares	1.090	0.763	1.134	0.865	1.260
11	Yanjing Beer	1.266	0.988	1.379	1.363	0.929
12	Shuanghui Development	0.783	0.571	2.188	1.250	0.626
13	Sanquan Food	1.290	1.774	0.560	0.993	1.300
14	Yanghe Shares	2.055	1.664	1.017	1.692	1.214
15	Delisi	1.103	0.094	2.193	0.207	5.335
16	Vivian Shares	3.847	0.086	1.761	0.152	25.260
17	COFCO Sugar	0.961	1.000	0.893	0.893	1.076
Average		4.581	1.405	1.172	1.647	2.782

*Source: Data Processing*.

**Figure 3:**
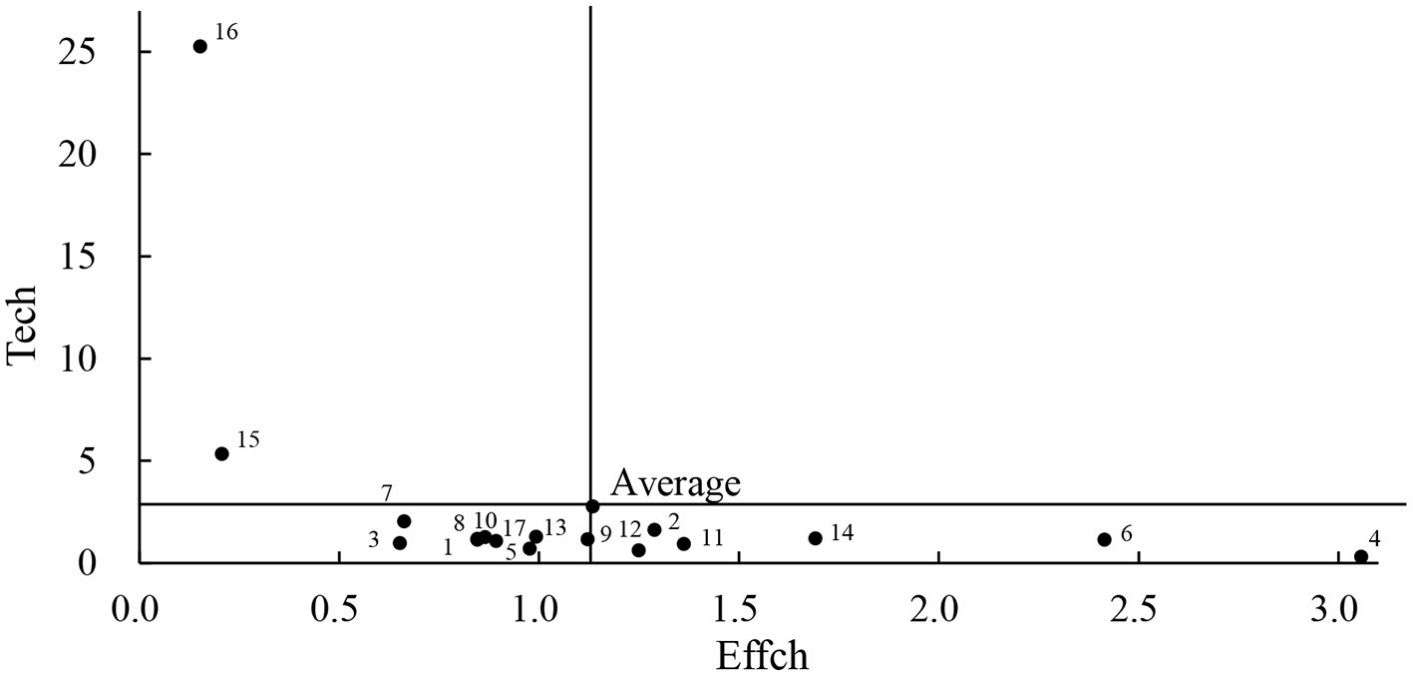
Composition of changes in comprehensive efficiency of the 17 sample enterprises in 2020.

The development within the enterprises in the food industry is uneven in terms of technical efficiency and technical progress. To further analyze the composition of total factor productivity change, technical efficiency change is used as the horizontal coordinate, technical progress is used as the vertical coordinate, and the average value of technical efficiency change, as well as the average value of technical progress change, is used as the threshold value, to divide the sample companies into four quadrants to evaluate the total factor productivity change status in 2020 (see Figure 3). As can be seen from figure, most companies are located in the third and fourth quadrants. None of the companies is distributed in the first quadrant, i.e., no company's technical efficiency and technical progress are both above the industry average. What is more, it shows that the level of technical efficiency and the technical progress in the food industry is high. Still, most of the enterprises are located below the industry average.

From the analysis of technical efficiency and technical progress, Chengde Lolo, Guizhou Maotai, Tsingtao Beer, Erie Shares, and Sanquan Food all achieved positive total factor productivity growth even though their TECHCH and EFFCH are lower than the sample mean. The common reason is that the acceleration of technical progress is obvious, compensating for declining technical efficiency.

### Analysis of Factors Influencing Corporate Social Responsibility Efficiency in the Food Industry Based on the Tobit Model

#### Variable Selection

The super-efficiency DEA and Malmquist index objectively reflect the social responsibility efficiency of the sample companies and the Tobit regression is selected to further explore the key influencing factors of CSR efficiency.

Based on the literature research and the analysis of influencing factors, this article selects five explanatory variables: enterprise size, corporate governance level, shareholding concentration, profitability, and listing years, from the internal governance structure and the nature of their capabilities. The specific indicators and descriptions are shown in Table 7. Among them, the measure of corporate governance level refers to the studies of Rechner and Dalton (22) and Jiraporn et al. (23), companies which meet the criteria that the chief executive officer (CEO) and Chairman of the Board are not concurrent and that there are four or more committees on the board are considered to have a high level of governance.

**Table 7:** Factors influencing CSR efficiency.

**Type**	**Variables**	**Symbols**	**Indicator description**
Explained variables	CSR efficiency	CSR	Corporate social responsibility efficiency
	Enterprise size	Size	Natural logarithm of total assets at the end of the year
	Corporate governance level	CG	Take 1 when the level of governance is high; otherwise, take 0
Explanatory variables	Shareholding concentration	Equity	Number of shares held by the largest shareholder/Total number of shares
	Profitability	Profit	(Current year's net profit - Prior year's net profit) / Prior year's net profit
	Listing years	Age	Current year - year of listing + 1, taking the natural logarithm

*Source: Literature Research*.

In this article, we believe that the size of an enterprise reflects its ability to assume social responsibility and the complexity of its internal operation process, which are closely related to its social responsibility efficiency. The degree of attention to social responsibility may vary. The level of corporate governance reflects whether the enterprise's shareholders, directors, and executives have their respective roles and responsibilities and its impact on the efficiency of social responsibility cannot be ignored. The concentration of shareholding directly reflects the shareholding structure of the enterprise and the shareholding of the major shareholders, which represents the stability of the enterprise's internal control and has a close relationship with the internal governance of the enterprise. The level of profitability reflects whether the enterprise's business activities have a solid economic foundation, which affects the ability and efficiency of the enterprise to fulfill its social responsibility in terms of corporate strategy and business policy. The listing years reflect the enterprise's overall strength and operational experience.

#### Descriptive Statistics and Correlation Analysis

Corporate social responsibility efficiency was used as the explanatory variable and each influencing factor was used as the explanatory variable. The results of descriptive statistics of each variable are shown in Table 8.

**Table 8:** Results of descriptive statistics of the Tobit model variables.

**Variable**		**Mean**	**Std. Dev**.	**Min**	**Max**	**Observations**
CSR	Overall	1.534	1.390	0.405	10.373	*N* = 170
	Between		0.752	0.538	3.256	*n* = 17
	Within		1.182	−0.994	8.652	T = 10
Size	Overall	23.349	1.219	20.816	26.086	*N* = 170
	Between		1.170	21.264	25.253	*n* = 17
	Within		0.436	22.110	24.897	T = 10
CG	Overall	0.906	0.293	0.000	1.000	*N* = 170
	Between		0.156	0.500	1.000	*n* = 17
	Within		0.250	0.006	1.406	T = 10
Equity	Overall	0.399	0.156	0.088	0.734	*N* = 170
	Between		0.150	0.108	0.608	*n* = 17
	Within		0.053	0.108	0.582	T = 10
Profit	Overall	−0.437	6.215	−73.115	8.835	*N* = 170
	Between		1.918	−7.309	0.604	*n* = 17
	Within		5.928	−66.243	9.563	T = 10
Age	Overall	2.643	0.530	0.693	3.332	*N* = 170
	Between		0.468	1.750	3.149	*n* = 17
	Within		0.271	1.586	3.291	T = 10

*Source: Data Processing*.

To test for multicollinearity among the explanatory variables, correlation analysis was performed on each explanatory variable included in the Tobit model and the results are shown in Table 9. The correlation of each explanatory variable was not overly significant, but there were still cases where the correlation coefficient was >0.5. Therefore, it was necessary to further test whether there was multicollinearity among the variables. The variance inflation factor (VIF) was obtained by testing the covariance of each explanatory variable and the VIF values were found to be <10, indicating that there was no serious multicollinearity among the explanatory variables.

**Table 9:** Results of correlation analysis and multicollinearity test.

	**Size**	**CG**	**Equity**	**Profit**	**Age**
Size	1				
CG	0.110	1			
Equity	0.069	−0.053	1		
Profit	0.101	−0.002	−0.028	1	
Age	0.508*	0.054	0.060	−0.019	1
VIF	1.390	1.020	1.010	1.020	1.360

Significance: ^*^p < 0.1.

*Source: Data Processing*.

#### Hausman Test and Model Building

The original hypothesis of the Hausman test is that the coefficients of a random-effects model do not differ from those of a fixed-effects model. If the original hypothesis is accepted, it indicates that a random-effects model should be chosen, otherwise a fixed-effects model should be chosen. We used Stata version 15.0 to test the model setting of the panel data by establishing a random-effects model among the variables and conducting the Hausman test. The test results are shown in Table 10.

**Table 10:** Results of the Hausman test.

	**Fixed effect**	**Random effect**	**Difference**	**S.E**.
Size	−0.440	−0.354	−0.086	0.253
CG	0.282	0.276	0.006	0.133
Equity	−2.812	−1.038	−1.774	1.486
Profit	−0.061	−0.059	−0.002	0.003
Age	1.115	0.620	0.495	0.364
CONS	9.709	8.299	1.410	5.232
chi2(6)			8.240	
Prob>chi2			0.221	

*Source: Data Processing*.

As shown in Table 10, according to the results of the Hausman test, the *p*-value is 0.221, indicating that the test results did not reject the original hypothesis, so the panel data of CSR efficiency influencing factors should be modeled by the Tobit regression with random effects.

Using a random-effects model of the Tobit regression, combined with the above established index system of influencing factors, the relationship model between CSR efficiency and various influencing factors of enterprises in the food industry is established as shown in the following equation:


(5) $$\begin{array}{l} CSR_{it}=\beta_{0}+\beta_{1}Size_{it}+\beta_{2}CG_{it}+\beta_{3}Equity_{it}+\beta_{4}Profit_{it}\\ \;+\beta_{5}Age_{it}+\varepsilon_{it}() \end{array}$$

where *CSR*_*it*_ denotes CSR efficiency of firm *i* in year *t*, β_0_ denotes the regression constant, β denotes the regression coefficient, and ε denotes the random error.

#### Tobit Regression Results and Analysis

Based on the Tobit model established above, we use Stata version 15.0 to load the data of the food industry enterprises' social responsibility efficiency and various influencing factor variables from 2011 to 2020 and conducts the Tobit regression to obtain the regression coefficient and other parameters of each variable. The results are shown in Table 11.

**Table 11:** The Tobit regression results of factors influencing CSR efficiency.

	**Coef**.	**Std. Err**.	**z**	***P***
Size	−0.353	0.144	−2.46	0.014**
CG	0.278	0.340	0.82	0.413
Equity	−1.017	0.974	−1.04	0.296
Profit	−0.059	0.015	−3.93	0.000***
Age	0.611	0.314	1.94	0.052*
CONS	8.296	3.000	2.77	0.006***

Significance: ^*^p < 0.1, ^**^p < 0.05, ^***^p < 0.01.

*Source: Data Processing*.

As shown in Table 11, in the Tobit regression model established above, profitability passes the test at the 1% significance level and enterprise size passes the test at the 5% significance level. Listing years pass the test at the 10% significance level, indicating that the above three variables are the key factors affecting CSR in the food industry. The corporate governance and shareholding concentration level did not pass the significance test, indicating that their effects on CSR efficiency are insignificant under this model.

The expansion of enterprise size will reduce CSR efficiency to a certain extent and the impact is more significant. In terms of enterprise size, the coefficient of the natural logarithm of total assets at the end of the year on CSR efficiency is −0.353. This evidence indicates that although the expansion of enterprise size will provide enterprises with greater ability to fulfill their social responsibility, it also tends to cause problems such as the complexity of internal operations or too many links, resulting in higher costs and lower CSR efficiency.

At the level of corporate governance, the independence of the general manager and chairman and the integrity of corporate committees positively impact CSR efficiency. This issue indicates that the effective monitoring, checks and balances, and decision-making role of the company's board of directors can help to improve social responsibility performance and efficiency. Still, its effect is not significant and is not a key influencing factor of CSR efficiency.

Corporate shareholding concentration, on the other hand, harms CSR efficiency, reflecting that in internal control, major shareholders ignore the efficiency of social responsibility input to a certain extent or there may be errors or deficiencies in relevant decisions, which is not conducive to the implementation of corporate social responsibility and the improvement of efficiency. However, the effect of this variable is also insignificant and does not have a critical impact on CSR efficiency.

Regarding profitability, the coefficient of return on assets on CSR efficiency is −0.059, a negative effect and highly significant. Generally speaking, the rise in profitability will help companies to improve their ability to fulfill their social responsibility. Still, there are cases that after making profits, companies will be more inclined to invest their profits in the expansion of their scale or other strategic purposes and continuously invest for the improvement of their competitiveness, while neglecting the implementation of socially responsible behaviors, thus causing a decline in CSR efficiency.

In terms of listing years, the influence coefficient of listing years on CSR efficiency is 0.611, which has a positive effect and a slightly significant influence. This result proves that when companies have been listed for a longer period, they are more rational in their capital investment, have more experience and stronger sustainability, and attach importance to CSR, thus promoting the improvement of CSR efficiency.

#### Tobit Regression Results and Analysis Under the Influence of the Pandemic

Considering that COVID-19 pandemic in 2020 has greatly impacted various industries, we will explore the changes and differences in the factors influencing the social responsibility efficiency of food industry companies before and after the pandemic outbreak. Also, based on the Tobit model established above, using the abovementioned social responsibility efficiency of food companies in 2019 and 2020 and the variable data of various influencing factors, respectively, represent the situation before and after the outbreak and perform the Tobit regression. The results are shown in Table 12.

**Table 12:** The Tobit regression results of factors influencing CSR efficiency under the influence of pandemic.

	**2019**				**2020**			
	**Coef**.	**Std. err**.	**t**	***P*** **> t**	**Coef**.	**Std. err**.	**t**	***P*** **> t**
Size	−0.963	0.534	−1.8	0.096*	0.044	0.224	0.2	0.847
CG	2.073	1.934	1.07	0.305	−0.382	1.116	−0.34	0.738
Equity	−1.958	3.513	−0.56	0.587	0.189	1.603	0.12	0.908
Profit	0.494	1.143	0.43	0.673	−0.082	0.112	−0.73	0.477
Age	0.468	2.027	0.23	0.821	−2.061	0.890	−2.31	0.039**
CONS	22.396	10.466	2.14	0.054*	6.897	4.281	1.61	0.133

Significance: ^*^p < 0.1, ^**^p < 0.05.

*Source: Data Processing*.

From Table 12, we can see that in 2019 before the pandemic outbreak, enterprise size and shareholding concentration negatively impacted CSR efficiency, while the level of corporate governance, profitability, and listing years has a positive impact, among which only enterprise size is the key influencing factor. In 2020 after the pandemic, corporate governance, profitability, and listing years, which were originally positive, all became negative effects. The enterprises' size and shareholding concentration change from negative to positive effects. Among them, the significance of enterprise size disappears and the listing years become the key influencing factor of CSR efficiency.

The above comparison illustrates that the arrival of COVID-19 has had a great impact on the food industry. Enterprises with larger scale and more stable internal controls are more likely to reduce their operations and continue fulfilling their corporate social responsibilities under the pandemic's impact. However, enterprises with higher levels of governance or higher profitability harm CSR efficiency, which may be because enterprises uniformly focus their resources on reducing the pandemic's impact and ignore the improvement of CSR efficiency. The cost of adjusting the internal structure and profit model is relatively high, which will hurt the efficiency of social responsibility. In addition, the number of years on the market became a key negative factor affecting CSR efficiency after the pandemic outbreak, probably because the impact of COVID-19 required enterprises to make timely adjustments to their existing models. Enterprises with longer listing years have accumulated more investment.

## Conclusion

Based on the background of COVID-19 and taking 17 enterprises in the food industry whose social responsibility performance is above the industry level from 2011 to 2020 as an example. After using the super-efficiency DEA-Malmquist-Tobit model to analyze the CSR efficiency of 17 food enterprises in China from 2011 to 2020, this article gets some conclusion, which are as follows:

First, according to the static analysis of the super-efficiency DEA model, the average value of CSR efficiency of 17 samples in the food industry from 2011 to 2020 is 1.534 and the overall level of CSR efficiency is high. On the whole, COVID-19 has promoted the social responsibility of the sample enterprises in the food industry, with individual differences in the impact on the food industry, among which enterprises with good CSR performance before the pandemic are more affected. There is an obvious gap in social responsibility efficiency among these enterprises. At the same time, among Chinese food enterprises with good social responsibility performance, some enterprises have inefficient social responsibility efficiency investments. Therefore, Chinese food enterprises' social responsibility investment efficiency needs to be improved.

In addition, according to the dynamic analysis of the Malmquist productivity index method, the average total factor productivity of 17 sample enterprises from 2011 to 2020 was 1.957 and the annual total factor productivity was >1. The overall CSR efficiency was on the rise year by year. From the perspective of technical efficiency change and the technical efficiency level of enterprises has been rising in the past 10 years, but the fluctuation trend is relatively large. The average scale efficiency has pulled down the growth level of technical efficiency. From the perspective of technological progress change, the changing trend of technological progress is generally consistent with total factor productivity. Therefore, technological progress is the main factor affecting the change of total factor productivity of corporate social responsibility. From the perspective of total factor productivity, the overall CSR efficiency of 17 sample enterprises in the food industry from 2011 to 2020 was jointly affected by changes in technical efficiency and technological progress. From the overall level, total factor productivity was more affected by changes in technological progress. Under the background of COVID-19, the level of technological efficiency and technological progress in the food industry is relatively high. Still, most enterprises are below the average level of the industry. The development of technical efficiency and technological progress within the food industry is unbalanced.

Finally, according to the Tobit regression analysis, enterprise size, profitability, and listing years belong to the key factors affecting social responsibility efficiency of food industry enterprises, among which, listing years have a positive effect on CSR efficiency. In contrast, enterprise size and profitability do not positively correlate with CSR efficiency. In addition, the level of corporate governance and shareholding concentration does not significantly affect CSR efficiency under this study and are not among the key influencing factors. The occurrence of COVID-19 pandemic, on the other hand, caused a great impact on the influencing factors of CSR in the food industry. Before the pandemic, enterprise size was the key influencing factor on CSR efficiency with a significant negative influence; after the outbreak, listing years became the key influencing factor with a negative relationship with CSR efficiency. The rest of the variables were not key influencing factors.

According to the above study conclusions, this article puts forward the following suggestions:

According to the principle of CSR, the laws related to the food industry should focus on safeguarding the interests of consumers to prevent damage to the interests of consumers due to legal loopholes. In terms of the government, the government should improve the laws and regulations on the social responsibility of China's food industry enterprises and give play to legal supervision and management. In addition, the government must hold public welfare lectures on social responsibility, publicize the knowledge of corporate social responsibility to consumers, change consumers' shopping concept, encourage consumers to pay attention to the performance of CSR, and the quality of food to guide the change of the atmosphere of the whole food industry.

In terms of the food industry, one of the reasons for the poor performance of social responsibility of Chinese food enterprises is the lack of unified social responsibility standards. Therefore, the Chinese food industry must setup corresponding social responsibility implementation standards according to their characteristics and gradually improve the statistical and quantitative systems of social responsibility indicators of food enterprises. Besides, it is necessary to reward enterprises with good performance in CSR within the industry, which will help to develop corporate social responsibility culture to improve the current situation of social responsibility of Chinese food enterprises.

For the efficiency of social responsibility investment, food enterprises should pay attention to various stakeholders' interests to achieve long-term and stable development, especially the interests of consumers, suppliers, and creditors. For total factor productivity, in the process of undertaking social responsibility, food enterprises should strengthen technological investment, introduce innovative talents, and increase the intellectual reserve of enterprises, which can improve the level of technological innovation and help enterprises to create unique high value-added products, find new profit growth points in the highly competitive market environment, to improve profitability and overall social responsibility efficiency.

## Study Limitations and Outlook

The study of CSR performance evaluation is a very applied field and this article also specific the research objects to food enterprises to emphasize this point. But, although this article combines theory and practice and builds a model that can be applied to the evaluation of the social responsibility performance of Chinese food enterprises, there are still some shortcomings in the depth of the study and the system of the study.

First, due to the study on CSR in China is still in the stage of growth and development, there are still many gaps in the social responsibility of enterprise, including food enterprises, so that the selected social responsibility input-output evaluation index in this article does not contain all the content of CSR. Therefore, in the further study, we will further explore and improve, make the study more representative.

Second, due to the limitations of objective conditions such as time and resources, the social responsibility performance evaluation model constructed in this article has not been empirically studied in specific food enterprises, so on the basis of the feasibility, its accuracy needs to be further tested by examples.

Third, enterprises of different nature have differences in the performance of social responsibilities. This article does not include this influencing factor into the construction category of CSR performance evaluation model in the food industry, which will affect the judgment of CSR behavior performance to a certain extent.

For the first limitation, this article believes that through the further exploration and research in the field of CSR and its performance evaluation in the future, we can obtain more and more representative indicators. For the second limitation, this article gives the following research idea: first, select a few food enterprises, which are representative and have research meaning and the specific information of the company is analyzed according to the established social responsibility performance evaluation model, so as to make the study more practical, rise from particularity to universality, and give study suggestions of universal significance. For the third limitation, this article will continue to carry out in-depth and rich study on the basis of the existing research and continue to build a scientific and reasonable food enterprise social responsibility performance evaluation model, to provide great significance for food enterprises and stakeholders from all the walks of life.

## Data Availability Statement

The original contributions presented in the study are included in the article/Supplementary Material, further inquiries can be directed to the corresponding author/s.

## Author Contributions

ZC and HF: writing original draft. WZ: reviewing the original draft. HL: writing original draft and data collection. All authors contributed to the article and approved the submitted versions.

## Conflict of Interest

The authors declare that the research was conducted in the absence of any commercial or financial relationships that could be construed as a potential conflict of interest.

## Publisher's Note

All claims expressed in this article are solely those of the authors and do not necessarily represent those of their affiliated organizations, or those of the publisher, the editors and the reviewers. Any product that may be evaluated in this article, or claim that may be made by its manufacturer, is not guaranteed or endorsed by the publisher.
